# Assessment of awareness and knowledge of schistosomiasis among school-aged children (6–13 years) in the Okavango Delta, Botswana

**DOI:** 10.1186/s41256-022-00267-x

**Published:** 2022-09-30

**Authors:** Kebabonye P. Gabaake, Nthabiseng A. Phaladze, Don Eliseo Lucero-Prisno III, Olekae T. Thakadu

**Affiliations:** 1 School of Allied Health Professions, University of Botswana, Gaborone, Botswana; 2 School of Nursing, University of Botswana, Gaborone, Botswana; 3 Department of Global Health and Development, London School of Hygiene and Tropical Medicine, London, UK; 4 Faculty of Management and Development Studies, University of the Philippines Open University, Los Baños, Laguna Philippines; 5 Okavango Research Institute, University of Botswana, Maun, Botswana

**Keywords:** Awareness, Knowledge, Schistosomiasis

## Abstract

**Background:**

Schistosomiasis is a global health problem affecting 250 million people, with 90% in Sub-Saharan Africa. In Botswana, the burden is high in the Okavango delta because of the water channels. WHO recommends integrated measures, including access to clean water, sanitation, health education, and drugs to control and eliminate schistosomiasis. Gauging knowledge and awareness of schistosomiasis for School-Aged Children (SAC) is crucial. Our study aimed at assessing knowledge and awareness of schistosomiasis among SAC in the Okavango Delta.

**Methods:**

A cross-sectional survey assessing awareness and knowledge of schistosomiasis in schools was conducted. 480 questionnaires were administered to gather demographic profiles, awareness, and knowledge of risky behaviors. Chi-square and descriptive analysis determined the differences in SAC`s awareness and knowledge levels based on localities, gender, age, and health education.

**Results:**

The results showed a low awareness level, with only (42%) of respondents having heard about the disease and (52%) knowing its local name. Younger children from Sekondomboro (83%) and Samochima lacked awareness, while children from Mohembo (77%) and those who had health education (70%) demonstrated significant awareness levels (*P* ≤ 0.001). Seventy-two percent (72%) lacked knowledge of the cause and (95%) did not know the disease life-cycle. Children from Xakao (91%), (85%) Sepopa, and (75%) of younger children did not know haematuria is a symptom of the disease. Older and SAC with health education were more likely to know that swimming is a risk factor (*P* ≤ 0.001) and (*P* ≤ 0.05) respectively.

**Conclusions:**

Although respondents from four schools demonstrated some level of awareness of the disease, and knowledge of risky behaviors, the study showed a lack of in-depth knowledge on the life-cycle and cause of the diseases. We, therefore, recommend the implementation of an integrated approach to health education and improvement in access to clean water and sanitation in all study areas.

## Background

Schistosomiasis is a Neglected Tropical Disease (NTD) with negative effects on public health. Endemic in 78 tropical and sub-tropical countries, schistosomiasis afflicts approximately 250 million people worldwide, with more than 90% of cases occurring in Sub-Saharan Africa [1–3]**.** An estimated 20,000–200,000 people die of schistosomiasis each yea**r **[3]**.** The disease is ranked among the top ten causes of disability in six African countries [4] with an estimated 3, 7900 Years of Life with Disability (YLDs) in 2012 attributed to schistosomiasis. The highest prevalence of the disease in Africa is seen in Nigeria (29 million) followed by Tanzania (19 million) and Ghana and the Democratic Republic of Congo both at 15 million [5]. School-Aged Children (SAC) bear most of the burden of this disease in Africa [5, 6]. Botswana bears an overall prevalence of 38% for Schistosoma. Haematobium in the Okavango region carries the highest burden of the disease [7]**.**

Schistosomiasis is a parasitic disease caused by blood flukes of the genius schistosome [8, 9]. Humans are usually infected by five species of Schistosoma mansoni, Schistosoma japonicum, Schistosoma mekongi, and Schistosoma intercalatum causing intestinal infection and haematobium responsible for urinary infection [10]. The sub-Saharan African infection is caused by Schistosoma mansoni *and* Schistosoma haematobium. The disease is often acquired during domestic, occupational, and livelihood activities which involve contact with water infested with cercariae from the intermediate host-the snail [5]. Climate change, ecological changes such as dam construction, and socio-economic factors have been crucial factors in the continuous transmission of schistosomiasis in endemic areas [5, 10]**.** In Botswana, the Okavango region presents a major threat of schistosomiasis because of its potential for a resurgence due to flood recession farming and the predicted increase in the seasonal flow of the delta [11, 12]**.** In their research, Appleton, and Madsen [13] indicated that hydrological factors seem to play an important role in disease transmission than livelihood activities in Botswana. The authors [13] noted that the population living near the river and children attending schools near the river in Botswana are at an increased risk of repeated epidemics. Recognizing the intense studies on the contribution of the aforementioned risk factors on the rate of infection with schistosomiasis, it is assumed that the complexity of the socio-economic, behavioral, and knowledge factors vary considerably at global, regional, and local levels, hence the need for in-depth risk factor analysis for their contextual understanding.

The World Health Assembly (WHA) has made several resolutions [14] and specified control and elimination measures of schistosomiasis. These include snail control, mass treatment, improved sanitation, provision of potable water, and health education. Disease control programs recommended for School-Aged Children (SAC) include Mass Drug Administration (MDA), sanitary methods, and health education. The WHO guidelines recommend every-other-year praziquantel treatment of all SAC in communities having moderate risk (prevalence between 10 and 49%), and annual SAC treatment for communities having high risk (prevalence ≥ 50%) [15]. Re-infection with Schistosoma haematobium among SAC is of global concern, especially with low (34.6%) MDA program coverage and low implementation of preventive therapy (40.8%) in the African region [16]. Several studies [17, 18] indicated that often after treatment new infection cases resurface to the point where baseline prevalence is reached within a short period. The 65.21 World Health Assembly resolutions [9] therefore called for the inclusion of all high-risk groups, including SAC, to eliminate schistosomiasis.

The schistosomiasis control program for Botswana was integrated into the Primary Health care services in 1983, and by 1985, the program existed in four districts of the country and was sustained until 1991 when Botswana reached elimination status. Despite, the huge success of the control program back then, Botswana still battles with transmission interruption and complete elimination of the disease, especially in the Okavango region. With a high-level prevalence of 38% [7] and impairing morbidity and the resultant complications, especially among the SAC, the disease has been undervalued in the past three decades in Botswana, thus qualifying it as a neglected disease. Moreover, the scarcity of data showing risk factors and their associated behaviours and rates of re-infections in Botswana poses scientific and ethical challenges in addressing the burden of this disease. Primary school-aged children in endemic foci are considered a high-risk group but there is a scarcity of data in Botswana showing the dynamics of re-infections among school children to inform current and future control strategies. Also, the Botswana schistosomiasis control program has heavily relied on the test and treats strategy, where cases detected through the Kato-Katz method are treated with 40 mg/kg of praziquantel [12]**.**

Communities in the Okavango have low levels of knowledge and awareness of the disease [19]**.** Adequate knowledge and adoption of correct and consistent preventive behavior in endemic areas are necessary for an effective and sustainable control program owing to the elimination of the disease. Although health promotion through community education is an important aspect of the elimination of any disease, a review of literature in this area for Botswana SAC revealed a paucity of empirical data owing to decadal neglect of the disease.

There is insufficient knowledge in Botswana which mapped the knowledge and awareness of schistosomiasis and its risk factors. This calls for assessment knowledge of key risk factors for infections within endemic communities. This study aimed at assessing awareness and knowledge of risk factors for schistosomiasis among SAC in the Okavango Delta. The specific objectives are to determine the SAC`s i) awareness of the disease, ii) general knowledge, and iii) knowledge of risky behaviors of schistosomiasis among SAC in the Okavango Delta.

## Methods

*Study design* This wasa cross-sectional survey that assessed students’ knowledge and awareness of schistosomiasis haematobium and its associated risky behaviours.

**Fig. 1:**
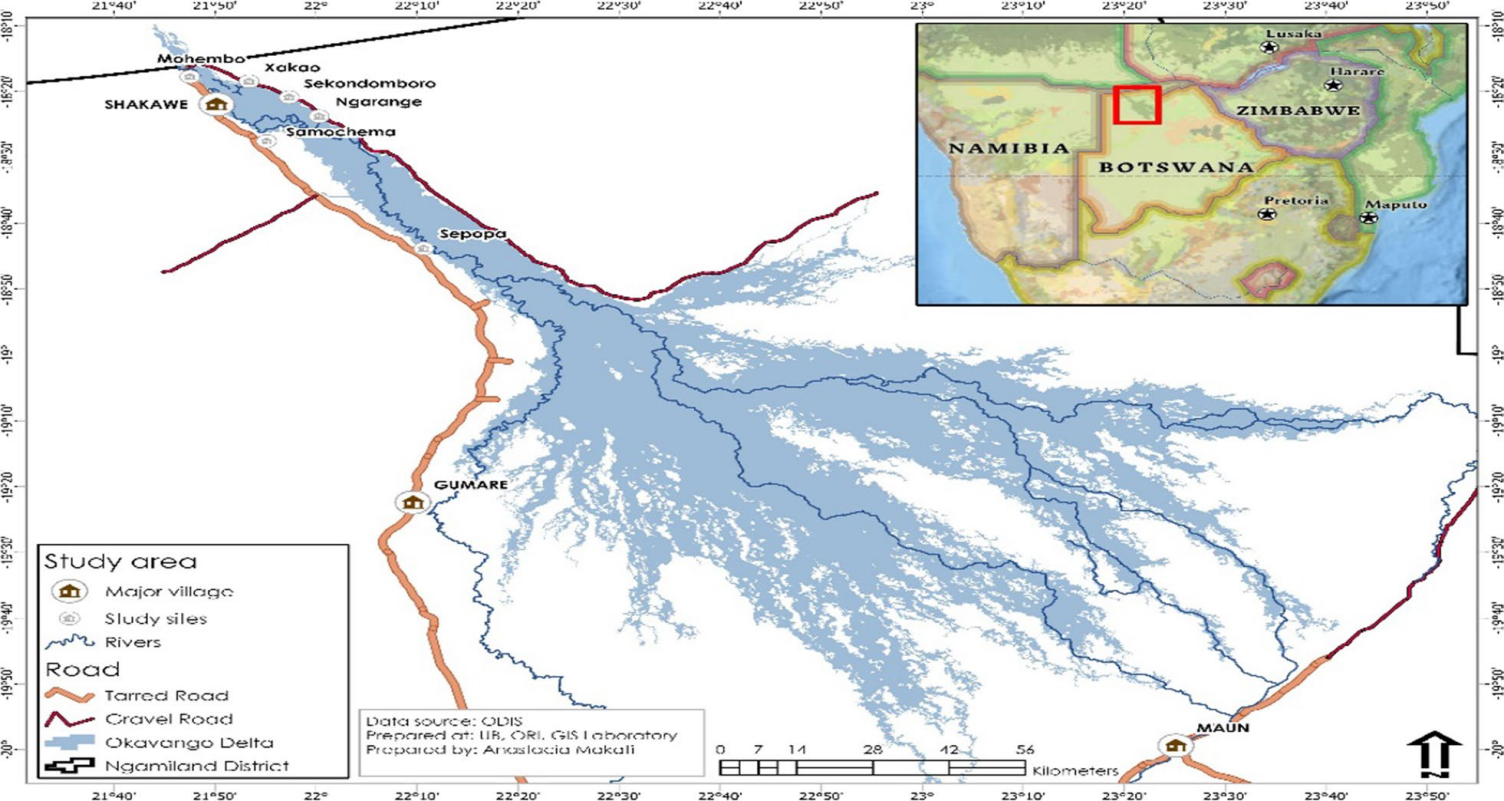
Map of the Okavango delta and study sites. ODIS (2019); “okavango delta information system”, University of Botswana, Okavango Research Institute from https://www.odis.ub.bw/portal/home

*Study setting* The study was conducted in the Ngamiland district in the Northwestern part of Botswana which has a population of 175,631 [20]**.** The site is part of the Okavango Delta which is one of the world`s largest wetlands. The delta comprises permanent, seasonal, and swampy areas which originate from upstream of Angola and Namibia. The Cubango River from Namibia enters Botswana and travels along with the stream passing into upper villages of the panhandle. As it travels along these villages it gives way to seasonal swamps. The flood is often at its peak at Mohembo between mid-March and mid-May immediately after the summer rains. The delta is the residence of a multi-ethnic group of people who include: Bayei, Hambukushu, San/Khoikhoi, and Batawana. More than 8000 people live in six villages along the Okavango River where the study was conducted (Fig. 1) [20, 21]. Six schools in villages along the Okavango watercourse were purposively sampled as sites for the study. Each village has one primary school. The proximity of the villages to the wetland and water contact livelihoods which often involve schoolchildren provided a good opportunity for studying schistosomiasis. The selected villages are proximal to the seasonal flood plains/permanent water channels status. The upstream schools of Samochima, Mohembo, Skondomboro, Ngarange, Xakao, and Sepopa were included in the study. Sepopa is one among the selected villages that are further at the mid-upper delta or where the Panhandle spread into the alluvial Delta.

### Study population and sampling strategy

**Fig. 2:**
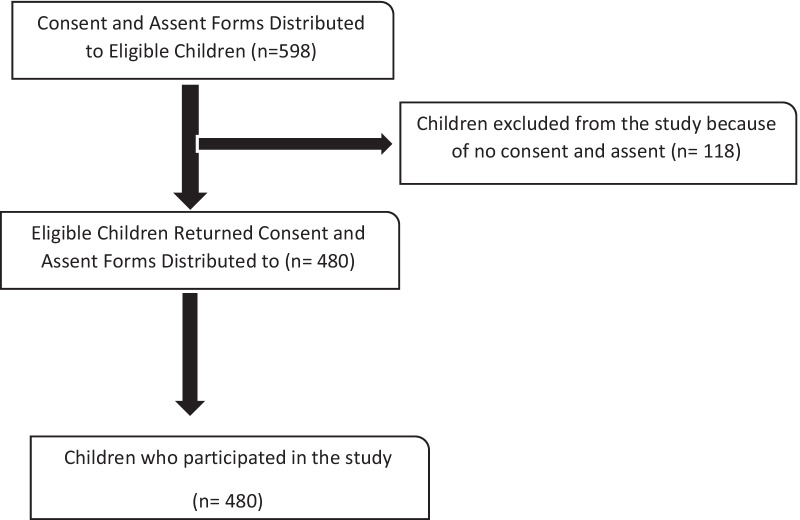
Study participant flow diagram

The sample for the study consisted of a population of 2417 children in standard 1 to 6, aged 6–13 years. Six Primary schools in the Okavango region were purposively selected to be included in the study due to their geographical location and being close to the river course. The sample size of 598 SAC was estimated using Yamane’s formula for the cross-sectional survey [22]**.** The eligible children were requested for assent and parental consent. One hundred and eighteen (118) of the 598 did not consent hence their exclusion from the study, (refer to Fig. 2). The response rate for the study was however notably high (80%).

A stratified random sample proportionate to size was drawn from each school as shown in Table 1. In each selected school, the total number of respondents per standard was obtained from the school head to determine the number required from each standard. The selection of children at each school was done through systematic random sampling using class lists obtained from class teachers and only 480 respondents participated in the study.

**Table 1:** Proportions of required samples

Villages	Total school enrolment	Required sample per school	Consented to the study
Sepopa	250	62	55
Samochima	394	97	93
Mohembo	494	122	101
Xakao	659	163	100
Skondomboro	268	66	63
Ngarange	352	87	68
Total	2417	598	480

### Survey questionnaire development and validation

A structured questionnaire was developed using the Theoretical Rational Approach (TRA), where targeted constructs of knowledge and awareness of schistosomiasis were adopted and adapted from literature and previous related research work [23]. The literature that guided the questionnaire construction included Xiao and colleagues` Protection Motivation Theory (PMT) [24] and the Knowledge Attitude and Practice (KAP) Theory [25–27].

Derived from previous similar studies [28–31]**,** 13 subjective measures on knowledge of signs and symptoms, key risky behaviours, and the lifecycle of the disease were constructed. The items on knowledge did not comprise a score but assessed if the respondents possess the correct information and knowledge about the disease. The scales comprised “yes, no, or I don’t know” and probes with pre-coded responses. Awareness measures were also developed based on previous related studies [32–35] and comprised closed-end (yes and no) and open-ended items.

The questionnaire consisted of three main sections. The first section comprised 8 items on the respondents’ socio-demographic information. The second section comprised 10 questions, 8 of which were closed-ended (yes, no and I don’t know) and 2 open-ended measuring awareness. Questions measuring knowledge were also part of this section and comprised 2 open-ended and 6 close-ended items. The last section comprised 7 questions on the respondents’ knowledge of key risky behaviours. The Cronbach’s alpha coefficient  for 8 close-ended questions on the domain of awareness was 0.81, indicating an adequate internal consistency. The Cronbach’s alpha score for the knowledge of risk factors items was almost 0.70 (0.69). While this is marginal, the mean inter-correlation for the items was 0.35, which according to Briggs and Cheeks is within the optimal ranges (0.2–0.4) that is recommended [36].

Pretesting in one non-selected school was done to assess content validity, appropriateness, and question comprehensiveness, and revisions were made based on the findings of the pre-test.

### Data collection

Data was collected on January 18–28th, 2020 using the questionnaire directly administered by the researchers. Community research assistants received training to assist with the data collection. Mini-sized cellular tablets which had the Research Electronic Data Capture (REDCap) software installed in them were used for data collection. The researchers were onsite to ensure appropriate data capturing and to address any technical issues. Two data collectors and the principal investigator used 4 electronic tablets to collect data across the six study sites.

### Data analysis

Data were exported from the REDCap and entered into Statistical Package for Social Scientist (SPSS) version 25.0 for management and analysis. Descriptive statistics (proportions and mean) were computed to summarise and describe the sample. Chi-squared tests of independence were used to determine the associations between demographic variables and variables of interest. Inferences using P ≤ 0.05 to test for statistical significance were made on the relationship between variables. All quantitative analyses were done at the 5% level of significance.

### Ethical consideration

The study design and protocol were reviewed and approved by the Human Research Development Committee at the Botswana Ministry of Health and Wellness (letter referenced HPDME: 13/18/1) and the University of Botswana Institutional Review Board (referenced UBR/RES/IRB/BIO/154). Parental consent was sought and children were asked for assent at the beginning of the interview.

## Results

### Socio- demographics profile of respondents

A total of 480 respondents (80% response rate) 6–13 years were enrolled in the study, of which 51% were females and 41% were males. The mean age for the respondents was 9.13 years with a standard deviation of 1.66. Respondents were further divided into two age groups: 6–9 who formed the majority 59% and 10–13 years who were 41%. Most (21%) of respondents were from Mohembo and Xakao villages. Twenty percent (20%) of the respondents were in the fifth standard and the lowest number (10%) of respondents were from standard one (Table 2). Almost one-fifth of the respondents (38%) reported having received health education about the disease from different sources, while the majority (62%) had not.

**Table 2:** Respondents` socio-demographic characteristics

Variable	Category	Frequency (n)	Percentage (%)
Gender	Female	245	51
	Male	235	49
Age Group	6–9	282	59
	10–13	198	41
Standard	1	48	10
	2	83	17
	3	85	18
	4	92	19
	5	97	20
	6	75	16
School	Mohembo	101	21
	Ngarange	68	14
	Samochima	93	19
	Sekondomboro	63	13
	Sepopa	55	12
	Xakao	100	21
Health education about disease	No health education	297	62
	Had health education	183	38

### Awareness of schistosomiasis among respondents

Majority of older respondents (10–13 years) (51%) were aware of Bilharzia compared to younger ones (6–9 years) (35%), and an almost equal proportion of females (42%) and males (41%) have heard about the disease. Over two-thirds of SAC in Sekondomboro (79%) and Samochima (69%) villages had not heard about the disease. The majority, (93%), of respondents who had no health education about the disease, indicated that they had not heard about the disease. A chi-square test conducted to determine if there were any significant differences among age groups and between those who received health education and those who did not, showed that older respondents and those with health education were more likely to have heard about the disease (*P* ≤ 0.001) and (*P* ≤ 0.001) respectively. A relatively higher proportion of respondents in the age group 10–13, (57%), and males (57%) knew the local name of the disease to be “bilharzia”. The majority of respondents who had health education (70%) knew the local name of the diseases than those with no health education (41%). Most respondents from Mohembo village (77%) demonstrated more awareness of the local name of the disease than those from other villages. A chi-square test indicated no significant differences in awareness of the local name of bilharzia between males and females in the four villages, (*P* = 0.09) and the age groups (*P* = 0.99). Respondents who had health education (*P* ≤ 0.001) were more likely to be aware of the local name of the disease. Similarly, respondents from Mohembo were more likely to be aware of the local name of the disease (*P* ≤ 0.001) than those from other villages.

The majority of younger respondents (75%) from, Sekondomboro (83%), and Samochima (80%) villages indicated that children from their schools lacked awareness of the disease. Similarly, respondents who had no health education about the disease (88%) were more likely to mention that other children from their schools lacked awareness of the disease than those who had health education. An almost equal proportion of respondents in the gender category indicate that other children from their school lacked awareness of the disease. Younger children (*P* ≤ 0.001), those who have no health education (*P* ≤ 0.001), and those from Sekondomboro and Samochima (*P* ≤ 0.001), were more likely to indicate that children from their schools lacked awareness of the disease. The results on the difference between males and females on this fact were not significant (*P* = 0.83), (refer to Table 3).

**Table 3:** Awareness of Schistosomiasis by demographic characteristics

Demographics	Frequency (n)		Heard about Bilharzia (%)		*P*-value	Know the local name of the disease (%)		*P*-Value	Think other children are aware of the disease (%)		*P*-Value
			No	Yes		Don’t Know	Bilharzia		No	Yes	
Age	6–9	282	65	35	0.001	51	49	0.09	75	25	≤ 0.001
	10–13	198	49	51		43	57		59	41	
Total	480	57	43			53	53		67	33	
Gender	Females	245	58	42	0.001	48	49	0.99	69	31	0.83
	Males	235	59	41		48	52		68	32	
Total	480	59	42		48	51		69	32		
Village	Mohembo	101	49	51	0.001	23	77	0.001	66	34	≤ 0.001
	Ngarange	68	47	53		47	53		52	48	
	Samochima	93	69	31		50	50		80	20	
	Sekondomboro	63	79	21		67	33		83	18	
	Sepopa	55	47	53		64	36		47	53	
	Xakao	99	59	41		53	47		73	27	
Total	480	58	42			51	49	67	33		
Health education status	No health education	297	93	19	0.001	59	41	0.001	88	12	≤ 0.001
	Had health education	183	11	89		30	70		35	65	
Total		480	54	52		45	56		62	39	

### General knowledge of schistosomiasis among respondents

The majority (72%) did not know that schistosomiasis was caused by parasites released during snail bites when one comes in contact with infested water. In terms of respondents’ knowledge of the effects of schistosomiasis on one’s health, a very small proportion (17%) and (23%) mentioned slow growth and poor concentration respectively. An overwhelming majority, (95%) of respondents did not know the life cycle of schistosomiasis in both humans and snails.

Concerning respondents’ knowledge of signs and symptoms of schistosomiasis, younger respondents (75%) did not know that blood in the urine (haematuria) is the main symptom of schistosomiasis compared with older children. The majority of respondents from Xakao (91%) and Sepopa (85%) and those with no health education (73%) did not know that haematuria is a symptom of schistosomiasis (see Table 4). The knowledge of fever as a symptom of schistosomiasis was very low (< 20%) across all the socio-demographics of the respondents. The results on fever as a sign were not significant between the age groups (refer to Table 4), of children who had and had no health education (*P* = 0.94). An overwhelming majority of older respondents (87%) than younger children (58%) did not know that lower abdominal pain is a symptom of schistosomiasis. Similarly, respondents from Xakao (99%), Sepopa (96%), and Mohembo (90%) didn’t know that abdominal pain was a symptom of the disease. A chi-square test for the difference in knowledge of abdominal pains showed that children from the three villages were more likely (*P* ≤ 0.001) to demonstrate a lack of knowledge of abdominal pain as a symptom than children from other villages. The results for the difference in knowledge of abdominal pain as a symptom between age groups (*P* = 0.29), gender (*P* = 0.75), and children with no or some health education (*P* = 0.97) were not significant.

**Table 4:** Knowledge of signs and symptoms of schistosomiasis by demographic characteristics

Socio-demographics	Frequency (n)		Fever (%)		*P*-value	Blood in Urine (%)		*P*-Value	Lower Abdominal Pain (%)		*P*-Value
			No	Yes		No	Yes		No	Yes	
Age	6–9	282	88	12	0.08	75	25	0.11	58	17	0.29
	10–13	198	82	18		54	46		87	13	
Total		480	85	15		65	36		73	15	
Gender	Female	245	85	15	0.08	69	31	0.001	85	15	0.75
	Male	235	86	14		63	37		84	15	
Total		480	86	15		66	34		85	15	
Village	Mohembo	101	81	13	0.02	75	25	0.001	90	10	0.001
	Ngarange	68	82	18		47	53		87	13	
	Samochima	93	75	25		35	65		61	39	
	Sekondomboro	63	87	13		60	40		78	24	
	Sepopa	55	93	13		85	15		96	4	
	Xakao	99	91	9		91	9		99	1	
Total		480	85	15		60	35		85	15	
Health education status	No health education	297	86	14	0.94	73	27	0.00	85	15	0.97
	Had health education	183	86	14		55	46		85	15	
Total		480	86	14		64	37		85	15	

### Knowledge of risky behaviors for the disease among respondents

The majority (71%) of respondents aged 6–9 years knew that swimming was a risk factor for schistosomiasis, with (81%) of those who had health education possessing knowledge. An equal proportion of females (76%) and males (77%) knew that swimming poses a risk for the disease. One hundred and seventy of the respondents (85%) who had heard about schistosomiasis, knew that swimming was a risk for the disease. The majority (81%) of respondents from Samochima and Xakao (79%) villages knew swimming was a risk factor for Bilharzia. A chi-square test showed that older respondents and those with health education were more likely to know that swimming as a risk factor (*P* ≤ 0.001) and (*P* = 0.04) respectively. Similarly, children who have heard about schistosomiasis were more likely to know swimming was a risk factor for the disease (refer to Table 5). There were no significant differences within villages between those who know that swimming is a risk factor and those who did not know (*P* = 0.12).

**Table 5:** Knowledge of swimming as a risk behaviour for Schistosomiasis by socio-demographics

Factor	Variable	Frequency(n)	Swimming (%)		*P*-Value
			No	Yes	
Age	6–9	282	29	71	≤ 0.001
	10–13	198	16	84	
Total	480	23	78		
Gender	Females	245	24	76	0.86
	Males	235	23	77	
Total		480	24	77	
Village	Mohembo	101	31	69	0.12
	Ngarange	68	15	85	
	Samochima	93	19	81	
	Sekondomboro	63	29	71	
	Sepopa	55	29	71	
	Xakao	100	21)	79	
Total		480	24	76	
Health education	No education	297	27	73	0.04
	Had education	183	19	81	
Total		480	23	77	
Heard about disease	Not heard	280	30	70	≤ 0.001
	Heard	200	15	85	
Total		480	23	77	

Children aged 10–13 years (71%) and those who received health education (74%) knew that walking barefooted in water poses a risk for schistosomiasis. Females (72%) were more knowledgeable about this fact than males (65%). The respondents who had received health education were more likely to know that walking in water barefooted was a risk factor for schistosomiasis (*P* = 0.03). There was no significant difference in age and gender (refer to Table 6) between those who did not know and those who know that walking barefooted in water was a risk factor for schistosomiasis Most (77%) of children who had heard about schistosomiasis knew that walking bearfooted in water was a risk for contarcting the disease compared to those who had not heard about it (63%). These results were significant (*P* = 0.001). Three quarters of children from Ngarange and Xakao (75% and 74% respectively) knew that walking barefooted in water was a risk for schistosomiasis. The comparison across villages were insignificant (*P* = 0.47) [see Table 6].

**Table 6:** Knowledge Walking in Water Barefooted by Socio-Demographics

Factor	Variable	Frequency (n)	Walking in water barefooted (%)		*P*-value
			No	Yes	
Age	6–9	282	33	67	0.39
	10–13	198	29	71	
Total	480	31	69		
Gender	Females	245	35	72	0.11
	Males	235	35	65	
Total		480	35	69	
Health education	No education	297	35	65	0.03
	Had education	183	26	74	
Total		480	31	70	
Village	Mohembo	101	35	65	0.47
	Ngarange	68	25	75	
	Samochima	93	32	68	
	Sekondomboro	63	37	64	
	Sepopa	55	36	64	
	Xakao	100	26	74	
Total		480	32	68	
Heard about disease	Not heard	280	37	63	0.001
	Heard	200	24	77	
Total		480	31	70	

The majority of female respondents (76%) considered drinking contaminated water as a risk for schistosomiasis compared to males (72%). Of the 198 respondents aged 10–13, (78%) mentioned drinking contaminated water was a risk for the disease compared to (71%) of those aged 6–9 years. Respondents from Samochima (59%) and Sekondomboro (60%) did not mention this factor as a risk for the disease. Respondents who had health education (83%) on the diseases mentioned drinking contaminated water as a risk factor to those who had no education (69%) of the disease. Health education (*P* = 0.01), and village (*P* = 0.001) were significantly associated with mentioning drinking contaminated water as a risk for the disease.

## Discussion

Active participation of local populations in prevention and control programs of schistosomiasis in endemic areas, is highly dependent on adequate knowledge and awareness of the disease, especially among children who are often the target of control programs. Our study findings show low levels of awareness of the disease, where only a few of the respondents had heard about the disease and just above half knew the local name of schistosomiasis. These findings corroborate those of other studies [37]**.** Respondents who had health education at least knew that haematuria was a sign of the disease. However, having health education did not influence knowledge of the signs and symptoms of the disease because our study records low levels of knowledge of the signs and symptoms. It is also important to note that the few respondents who had heard about the disease got information from school, while the health sector's role in educating children was very insignificant. This may be attributed to the decadal neglect of the disease since 1993 when the national control program was terminated. A high proportion of respondents had no health education on the disease. This shows that health education as a control strategy has not been implemented for over two decades. Our results are similar to a Yemen study [38]**,** which reported a lower knowledge of signs and symptoms but a higher level of awareness. A systematic review of the knowledge, attitude and practices on schistosomiasis found that knowledge of signs and symptoms is often low [39]**.** This poses challenges and threats to the uptake of most prevention and control strategies.

Our study found that males and those who had health education significantly knew haematuria as a symptom compared to females and those without health education. This could be explained by the fact that the universal local name for urinary schistosomiasis in Botswana is *“thuti-sa-madi"* which means blood in the urine. This finding is similar to that obtained in Swaziland [2] where 74% mentioned haematuria as a symptom. Again Folefac and collegues [40] in their study, recorded that among respondents who had prior knowledge of the disease, 80% mentioned haematuria as a symptom. With regards to males possessing more knowledge than females, a systematic review in sub-Saharan Africa [39]**,** found eight out of twelve studies that reported high levels of knowledge among males compared to females. The authors of a review [39] attributed better knowledge among males to health education provided to fishermen and farmers because of their exposure to risky water practices. The difference between the review and the Cameroon study [40] and our study is that our respondents were SAC while the two studies focused on adults.

Our study also revealed that respondents do not know the cause and the life cycle of schistosomiasis. This finding is similar to what Swaziland's [2] study obtained. However, Kitalile [41] reported a higher proportion (77.6%) of respondents who knew the cause of schistosomiasis. The difference may be that we asked closed-ended questions which limited exploration of possible responses which could have unearthed the real causes of the disease. Part of these differences might have been related to the fact that these studies used a different study method (that is, non-probability purposive sampling technique) than ours [42]**.**

Regarding the mode of transmission of the disease, which is indicated as a proxy from the question on drinking contaminated water, our study found some misconceptions from the respondents. A significant proportion of pupils across all villages mentioned that drinking contaminated water poses a risk of contracting schistosomiasis. This can be attributed to the fact that residents in the Okavango area often depend on contaminated river water for domestic purposes [43]**.** Consequently, outbreaks of water-borne disease (diarrhea) are common, especially during the dry season [44]**.** In addition to this, it is worth noting that the recurring unavailability and unreliability of water due to nonfunctioning taps and pumps and leaks experienced by villages in the Okavango delta, often prompt the utilization of river water for domestic use even in schools [45]. This may have prompted the respondents in our study to believe that schistosomiasis may be caused by drinking contaminated water. Moreover, this could suggest the lack of integrative approaches toward schistosomiasis and STH programming. Of greater concern is how these fears translate into preventive practices.

Regarding the knowledge of risk behaviours for schistosomiasis, our study found that younger pupils (aged 6–9 years) were more likely to know that swimming is a risk for the disease. This may be the influence of the teaching of content on schistosomiasis which is done at lower than upper school grades. Moreover, the health education in schools during MDA for STH in the past two years has contributed to this finding. Other studies [39] concurred that programs such as MDA improve awareness, knowledge, and uptake of preventive chemotherapy.

Notwithstanding the important findings from our study, we acknowledge the following limitations encountered during the implementation of the study; firstly, there was an issue with the subjectivity of responses. The self-reported data obtained from respondents lacked objectivity. Secondly, the study did not provide in-depth information about reasons for not having knowledge about schistosomiasis. It is important to note that being a cross-sectional survey, the study could not establish causal factors for the lack of knowledge and awareness which could be provided by analytic designs. There is a need, therefore, for a future qualitative and more analytic research design for this purpose.

The small sample size although adequate for statistical analysis used in the study, restricted the generalization of findings to the population, because the study was conducted in one sub-district, and hence may not be generalised to other sub-districts and districts elsewhere. Future studies must therefore target more districts using random sampling procedures for generalisation across the country. Not withstanding these limitations, the study results provided insights in knowledge and awareness of schistosomiasis among schools within the Okavango sub-district, which in turn informs and guided the following conclusions and recommendations.

## Conclusions

This study found that school children in the sampled schools of the Okavango delta lack awareness and knowledge of schistosomiasis with the majority not knowing the life cycle and most of the symptoms of the disease. This provides a window of opportunity to re-instating prevention and control measures of the disease within the area. SAC demonstrated a fairly average knowledge of water contact and risky behaviors of swimming and walking barefooted in infested water. Health education has impacted positively on the children’s awareness and knowledge, but other socio-demographics of age and gender did not. This is a signal of the importance of health education as a key component of the control program for schistosomiasis. There is thus the need for intense integration of school-based health education to improve the level of knowledge and awareness to reduce children’s water contact behaviors. Further research focused on unearthing reasons for low levels of awareness and knowledge is required to inform policy on elimination. More consideration and appreciation of the socio-cultural and cognitive behaviors of children than a focus on their age, gender, and place of residence which are found to have very little influence on their knowledge of risk behaviours for schistosomiasis prevention in the present study.

## Data Availability

The datasets used and/or analyzed during the current study are available from the corresponding author upon reasonable request.

## Abbreviations

MDA: Mass Drug Administration
NTD: Neglected Tropical Disease
SAC: School-Aged Children
STH: Soil-Transmitted Helminths
WHA: World Health Assembly
WHO: World Health Organization
